# Northern Indiana Dental Society

**Published:** 1890-01

**Authors:** J. E. Waugh

**Affiliations:** Fort Wayne


### CORRESPONDENCE.

FORT WAYNE, Oct. 12, 1889.

EDITOR REGISTER.

*Dear Sir*: The Northern Ind. Dental Society held its first meeting in the city of Fort Wayne last May, its second at Warsaw on the 17 and 18 of September, and the next meeting is to be held at Goshen on the first Tuesday and Wednesday of next May.

We elect a president, vice-president, and secretary at each meeting. The president appoints an executive committee whose duty it is to make all arrangements for the next meeting. We have no written constitution or by-laws and code of ethics.

Every dentist in the northern part of the State is considered a member, whether he attends or not, and always receives an invitation to be present.

Our last meeting was quite a success in point of interest and also in attendance. We confidently expect to have a still better meeting at Goshen.

The officers for the next term are: T. A. Goodwin, of Warsaw, President; B. P. McDonald, of Goshen, Vice-President; J. E. Waugh, of Fort Wayne, Secretary.

Executive Committee: B. P. McDonald, of Goshen; C. W. Leak, Goshen; S. B. Hartman, Fort Wayne.

Very Res'ly, J. E. WAUGH.

